# Assessment of seizure duration and utility of using SedLine^®^ EEG tracing in veterans undergoing electroconvulsive therapy: a retrospective analysis

**DOI:** 10.1186/s44158-024-00143-9

**Published:** 2024-02-06

**Authors:** Houman Amirfarzan, Kaitlin Jane Cassidy, Mehrak Moaddab, Ma Demin, Roman Schumann, Bradford Lewis

**Affiliations:** 1 Department of Anesthesia, Critical Care and Pain Medicine, VA Boston Healthcare System, Tufts University School of Medicine, Boston, MA USA; 2 Cooperative Studies Program Clinical Trials Coordinating Center, VA Boston Healthcare System, Boston, MA USA; 3 Department of Anesthesia, Critical Care and Pain Medicine, Tufts Medical Center, Tufts University School of Medicine, Boston, MA USA; 4 Department of Psychiatry, VA Boston Healthcare System, Harvard Medical School, Boston, MA USA

**Keywords:** Anesthetic IV, Methohexital, Electroconvulsive therapy, Processed EEG, Depth of anesthesia, SedLine^®^

## Abstract

**Background:**

Electroconvulsive therapy (ECT) endures as a definitive treatment for refractory depression and catatonia and is also considered an effective treatment for a number of other severe psychiatric disorders (Lisanby, N Engl J Med 357:1939-1945, 2007)(Weiner and Prudic, Biol Psychiatry 73:105-106, 2013). GA is an essential component of the ECT procedure for various reasons (Lee, Jenkins and Sparkle, Life 11, 2021). Monitoring anesthetic effects on the brain is desirable as anesthetic agents affect seizure duration and recovery (Rasulo, Hopkins, Lobo, et al,  Neurocrit Care 38:296-311, 2023) (Jones , Nittur , Fleming and Applegate,  BMC Anesthesiol 21:105, 2021) (Soehle , Kayser , Ellerkmann and Schlaepfer,  BJA 112:695-702, 2013). Perioperative anesthetic effects on consciousness can be assessed with brain function monitoring using raw electroencephalogram (EEG) traces and processed EEG indices.

**Objective:**

We examined the usefulness and utility of the SedLine^®^ anesthetic effect monitor during ECT procedures. We hypothesized that the seizure duration as measured by the EEG tracing of the ECT machine is equivalent to the duration assessed by the SedLine^®^ EEG tracing. A secondary objective was to describe the SedLine^®^ patient state indices (PSI) at different phases of treatment.

**Methods:**

Following IRB approval, we analyzed the data of the electronic medical records of 45 ECT treatments of 23 patients in an urban VA medical center between July 01, 2021, and March 30, 2022. We compared the seizure duration in minutes and seconds as measured either by the ECT machine EEG tracing or the SedLine^®^ EEG tracing. We then collected SedLine^®^ processed EEG indices at four different stages during the treatment. Appropriate comparative and observational statistical analyses were applied.

**Results:**

There was no significant difference in measured seizure duration between the two methods examined (*p* < 0.05). We observed a lag of the SedLine PSI value at the time before stimulus delivery and limited PSI utility during the course of ECT.

**Conclusion:**

The SedLine^®^ EEG tracing can be an alternative to the machine EEG tracing for the determination of seizure duration. The SedLine^®^ processed EEG indices are not consistently useful before and after ECT delivery. Anesthetic effect monitoring during ECT is feasible.

## Introduction

Electroconvulsive therapy (ECT) endures as a definitive treatment for refractory depression and catatonic states [1–3]. It is also widely considered a safe and highly effective treatment for a number of other severe psychiatric disorders, including those on the psychotic and bipolar spectrum [4]. Veterans are disproportionately affected by mental health disorders [5], most notably refractory depression and suicidality, and ECT is an indispensable treatment option for many of them.

The targeted neuronal depolarization of the brain induced by pulsatile electrical neurostimulation during ECT is associated with antegrade amnesia and, in the absence of neuromuscular blockade (unmodified ECT), tonic-clonic motor activity. The latter historically resulted in injuries such as tongue bites, dental injuries, and bone fractures [6, 7], and this is the predominant indication for general anesthesia (GA) including neuromuscular blockade (NMB) during ECT.

Raw and processed frontal EEG monitoring to assess the level of consciousness and anesthetic effect is no longer uncommon in the general surgical population but not yet a practice standard for anesthesiologists.

The SedLine^®^ Brain Function Monitoring system displays four real-time simultaneous frontal EEG waveforms, and in contrast to Bispectral Index^™^ (BIS), monitoring during ECT uses bilateral signals to quantify the level of consciousness [8]. The SedLine^®^ Brain Function Monitoring system calculates a patient state index (PSI). The PSI scale ranges from 0 to 100, with a number between 25 and 50 indicating adequate general anesthesia level, unconsciousness, and prevention of awareness. Before the PSI value is displayed on the monitor, the PSI is post-processed with an averaging algorithm, which provides a more stable output [9]. The BIS values correlated with depth of sedation in previous literature.

To our knowledge, there is no report on SedLine^®^ monitoring during ECT, and we are not aware of any studies that previously assessed the significance of the SedLine^®^ processed PSI values during various stages of the ECT procedure. The seizure time recording has not been mirrored to the seizure tracing of the ECT EEG machine used by the mental health team. Previously, the BIS failed to show any correlation with seizure duration [10]. We hypothesized that the seizure duration as measured by the EEG tracing of the ECT EEG machine is equivalent to the duration assessed by the SedLine^®^ EEG tracing. Our secondary objective was to describe the SedLine^®^ processed PSI numbers at different phases of treatment.

## Methods

Following IRB approval, we retrospectively collected anesthesia and ECT-related data from the electronic medical and anesthesia record as well as data from the SedLine^®^ Brain Function Monitoring system from 23 veterans who underwent ECT between July 01, 2021, and March 30, 2022. All records that documented the use of the SedLine^®^ Brain Function Monitoring system were included. Anesthesia-relevant data included type and dose of IV induction and NMB agents, and GA was administered per the routine standard of care at the institution. All patients were equipped with ASA standard monitors, and routine anesthetic agents were administered (Table 1). The induction agents included oxygen by mask, methohexital 1–1.5 mg/kg IV for 44 procedures, and 10 mg of etomidate for one patient [11]. Neuromuscular blockade was achieved by either succinylcholine 1–1.5 mg/kg IV or rocuronium 40 to 100 mg IV. Blood pressure, pulse oximetry, and capnography were recorded prior to treatment and 1 min, 3 min, and every 5 min following the ECT stimulus until the end of anesthesia care.

**Table 1:** Patient characteristics and co-administered medications (*N* = 23) in 45 treatment sessions

**Characteristics**	***n* (%)** **Mean [SD]**
Age	64 (15)
Gender	
Male	42 (89)
Female	5 (11)
Dexterity of patient	
Right	43 (96)
Left	2 (4)
Lead placement	
Bilateral	38 (84)
Unilateral	7 (16)
Anesthetic	
Methohexital	44 (98)
Etomidate	1 (2)
Anesthetic dose	
Methohexital dosage	101 (range: 70–220 mg)
Etomidate dosage	10 mg
Muscle relaxant	
Succinylcholine	40 (89)
Rocuronium	5 (11)
Muscle relaxant dose	
Succinylcholine dosage	104 (range: 100–140 mg)
Rocuronium dosage	48 (range: 40–100 mg)

### Using SedLine® and the ECT EEG machine

**Fig. 1:**
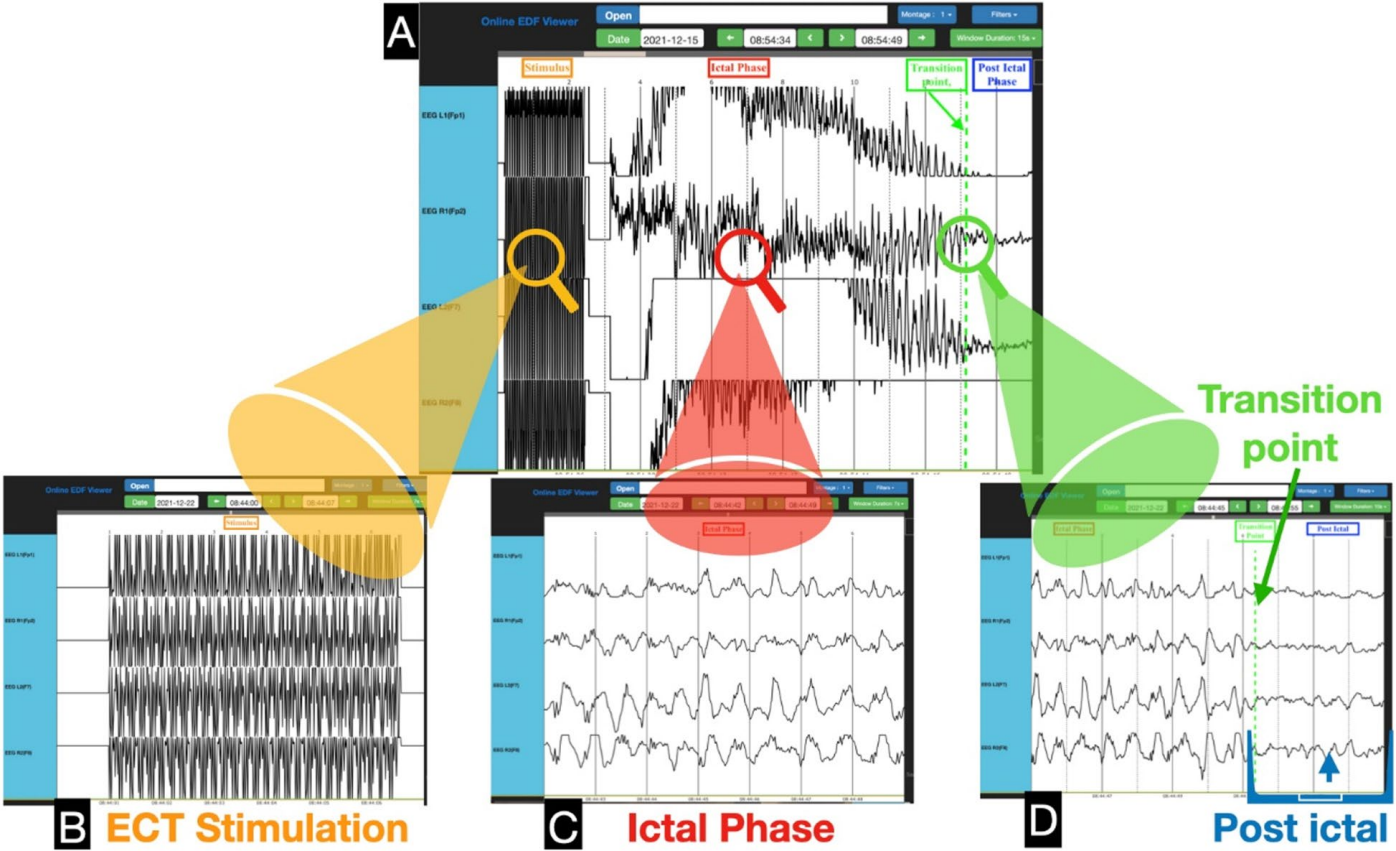
The EEG tracings were assessed with an EDF viewer software. **A** The four EEG channels include all the phases of ECT stimulation, ictal, and post-ictal phase. By slowing the tracing speed or shortening the view window, the EEG waveforms are better depicted. The zoomed windows are comparable to what is seen on the SedLine^®^ screen. **B** The EEG waveforms during ECT stimulation. **C** The ictal phase post ECT stimulation. **D** The moment of transition from ictal phase to post-ictal is readily observable in all four channel SedLine tracings. The transition point is demonstrated by a green line

We collected the following data: patient age, sex, and dexterity. Brain neuronal depolarization time (seizure duration) in minutes and seconds was determined from the start to the end of the neuronal depolarization of EEG waves on the ECT EEG machine and the SedLine^®^ Brain Function Monitoring system respectively. PSI data from the SedLine^®^ Brain Function Monitoring system data during different phases of treatment included the following: (1) baseline PSI (before anesthetic induction), (2) pre-ECT PSI (immediately before electrical stimulus delivery), (3) immediately after the end of neuronal depolarization (post-ECT) as determined by the mental health team ECT EEG machine, and (4) recovery PSI within approximately 10 min after leaving the ECT suite when clinically following any simple commands (eye opening in response to verbal stimulation). The time course of the raw EEG was exported to European Data Format (.edf) files for comparative analysis (Fig. 1).

### ECT

Depolarization and resulting neurostimulation were achieved utilizing either right unilateral or bitemporal stimulus-lead placement with a MECTA Spectrum 5000Q ECT device (MECTA Corp., Portland, OR, USA) according to the institutional standards of the mental health professionals. An on-screen and printed 2-lead EEG is a standard feature of all METCA Spectrum devices, and the printed EEG was used as the ECT EEG for all cases. Frontal EEG electrode placements was per mental health team routine care in the setting of ECT, and use of the SedLine^®^ monitor occurred as per user instructions with accommodation for the stimulus leads.

### Statistical analysis

Since our analyses were retrospective, power calculations were not computed, and the sample size was defined as the available number of records of patients who underwent ECT treatment with EEG recordings. Patient characteristics and ECT session data were entered into SAS version 9.4. (SAS Institute, Cary, NC, USA).

Normality and heteroscedasticity of the data were evaluated using multivariate normality plots. When appropriate, stabilizing transformations were applied to the data. Missing values were assessed graphically to determine if the data elements were missing not at random. Minimal data were missing, with the exception of two patients without SedLine PSI information that were removed from analyses. Frequencies, percentages, box and whisker, and residual plots were used to identify influential high leverage values. Goldilocks method of rounding was used (two significant figures versus decimal places) [12]. Patient characteristics, medications, and basic ECT data are reported in means, standard deviations (SD), counts, and percentages.

To compare seizure times measured with SedLine^®^ and the ECT EEG device in minutes and seconds (mm:ss), an equivalence test was performed. The absolute value of the difference between the recorded seizure times was used with *∆* set at 10 s. Equivalence bounds were computed by ±∆ with the mean (lower equivalence bound = −7:45 s, upper equivalence bound = 13:45 s). Devices were declared to be equivalent for differences < 10 s, and not equivalent otherwise.

Baseline PSI numbers at different stages during the ECT are reported as means (SD), medians, ranges, and 95% confidence intervals (CIs).

Linear relationships between age and electrical seizure duration with post-ECT PSI were examined using linear regressions. In the first model, age was used as the predictor variable and post-ECT PSI as the outcome variable. For the following two models, post-ECT PSI was the predictor variable, while motor and electrical seizure duration were outcome variables. *R*^2^ was computed to determine the variability of the outcome explained by the predictor within the data. Cook’s distance was used to determine existing influence points, while residual plots were generated to examine possible outliers.

To determine if recovery PSI is different among patients that had a bilateral versus unilateral stimulus placement, a two-sample *t*-test was performed. The Satterwhite correction was applied when variances were not equal. Unless indicated otherwise, *p*-values (two-sided) less than 0.05 were considered significant.

## Results

Documentation of 45 ECT procedures in 23 patients fulfilled inclusion criteria for this retrospective chart review. The average age of patients was 64 (±15) years. The majority of the patients were men (89%), right handed (96%), had bilateral stimulus placement (84%), and received methohexital as the IV anesthetic (98%) (Table 1).

### Seizure time comparison monitor

Neuronal depolarization time (seizure duration) was available for 45 treatments. The average seizure time for patients recorded on the SedLine^®^ device was 1:08 (±0:17) mm:ss and on the ECT EEG device 1:08 (±0:15) mm:ss. The average difference between the seizure duration recorded between the devices was 0:03 (±0:05). The 95% confidence interval (CI) for the difference was within the lower and upper bounds of the equivalence test (−7:45 < 0:02, 0:05 < 0:13) and was significantly equivalent (*p* < 0.0001).

### PSI values during various stages of the ECT

Availability of PSI data was variable at different stages of the ECT. The average baseline PSI value was 91 (*SD* = 8.5), 95% *CI* 89–94. The average pre-ECT PSI value was 74 (*SD* = 14), 95% *CI* 67–81. The average post-ECT PSI number was 40 (*SD* = 16), 95% *CI* 35–45. Post-ECT PSI was missing for four treatment records. The average recovery-PSI value was 64 (*SD* = 25), 95% *CI* 56–72 (Table 2).

**Table 2:** Summary of PSI measures at various time points in an ECT session

PSI	*N*	Mean (SD)	Median	Range	95% *CI*
Baseline	45	91 (8.5)	93	(40–100)	(89, 94)
Pre-ECT	19	74 (14)	76	(41–93)	(67, 81)
post-ECT	43	40 (16)	34	(19–87)	(35, 45)
Recovery	43	64 (25)	77	(20–88)	(56, 72)

### Age, motor seizure duration, electrical seizure duration, and ECT PSI

For the model examining the association between post-ECT PSI and age, a weak-positive linear association with an estimated slope of 0.16 (*SE* = 0.17) was observed. This means on average a one unit increase in age among patients receiving ECT corresponds to a 0.16 increase in post-ECT PSI. Age accounts for 2.1% of the variability of post-ECT PSI. The association between age and post-ECT PSI was not significant (*p* > 0.05). The model comparing post-ECT PSI and electrical seizure duration as the outcome showed a weak negative linear association with a slope estimated at 0.28 (*SE* = 0.21), meaning, on average with a one unit increase in post-ECT PSI, we can expect a 0.28-s decrease in seizure duration. The post-ECT PSI accounts for 4.2% of the variability of the electrical seizure duration. The association between post-ECT PSI and electrical seizure duration was not significant (*p* = 0.19). A weak negative linear association with an estimated slope of 0.21 (*SE* = 0.19) was seen for the model with pre-ECT PSI as the predictor and motor seizure duration as the outcome variables. This means that a one unit increase in PSI at ECT corresponds to a −0.21-s decrease in motor seizure time among patients. Pre-ECT PSI accounts for approximately 0.054% of the variability of motor seizure duration among these data. The association between PSI at ECT and motor seizure duration was not significant (*p* > 0.05).

### Unilateral ECT administration compared to bilateral ECT resulted in a lower recovery PSI

The mean recovery PSI following a bilateral stimulus placement was 69 (±22, *n* = 36), whereas the mean recovery PSI for unilateral stimulus placement was 41 (±29, *n* = 7). There was no significant evidence that the PSI variances were different (*F* = 1.7, *df* = 6, 35, *p* = 0.33). Therefore, we used the pooled formula for the 95% CI for the mean difference of PSI at recovery (8.3, 47).

Based on the *t*-statistic (assuming equal variances) of 2.9, with *df* = 41, there was a significant difference between mean recovery PSI after bilateral (69) and unilateral (41) stimulus placement respectively (*p* = 0.0063).

While not formally examined, during the care of these patients, we observed that the recovery PSI may indicate a PSI in a range reflecting anesthetic suppression of consciousness, while patients actually were clinically awake.

## Discussion

The results of our study indicate that the SedLine^®^ EEG tracing can be a valuable addition to the ECT EEG machine tracing for seizure duration assessment during ECT and confirms the accuracy of the SedLine^®^ EEG brain waves in this regard. An accurate alternative to the ECT EEG machine tracings used by the neuromodulation team for seizure duration assessment expands the objective neuronal depolarization measurement portfolio and provides a backup device for these patients.

Regarding anesthetic effect monitoring during ECT procedures using the PSI number of the SedLine^®^, we report several observations.

The pre-ECT PSI immediately prior to the stimulus delivery ranged between 41 and 93. A target number to achieve general anesthesia is < 50. Previous studies demonstrated the usefulness of anesthetic effect monitoring using the BIS^™ ^[13–15]. A higher BIS^™^ value is positively correlated longer neuronal depolarization and may indicate that less anesthetic was used in monitored patients than those without such monitoring [16, 17]. A lighter yet sufficient level of unconsciousness as guided by BIS^™^ monitoring provided superior neuronal depolarization characteristics [14, 16]. Deeper levels of unconsciousness impede seizure induction due to the anticonvulsant effect of anesthetics and probably will increase the risk of cognitive side effects [17, 18]. In our study, there was a trend that age will increase the PSI number. This aligns with a recent retrospective study finding that the patient’s age significantly influences both PSI and SEF (signal edge frequency) [19]. Previous studies with the BIS monitor also showed an index increase with age [20]. The PSI number generation lags behind the real-time tracings displayed on the SedLine^®^ monitor which are a highly reliable indication of the brain functional state. This delay time by SedLine^®^ monitor is reported by Obert et al. [19] and is estimated to be more than 50 s. This is longer than qCON and BIS delay that is approximately 25 s [21, 22]. Therefore, it is important for the treatment team to be familiar with the interpretation of frontal brain waves in concert with clinical patient assessment during teatment to prevent SedLine^®^ PSI data misinterpretaion. The time lag for PSI number generation most likely explains the high readings recorded for the pre-ECT PSI.

The immediate post-ECT PSI number is highly variable between treatments and is influenced by several factors including the parameters of the ECT stimulation protocol [23–28] and the anesthetic administered. The PSI number was between 19 and 87. It is entirely speculative whether or not the PSI at this time relates to the level of unconsciousness as the SedLine^®^ was not designed in the setting of a post-neuronal depolarization state in the brain. Hence, this number at this time may reflect the combined effects of residual anesthetics and a post-depolarization neuronal resting state. The PSI at this time should not be interpreted for level of consciousness assessment until further study clarifies its significance at this time point during treatment. Furthermore, our study showed a correlation of increasing age with increasing PSI immediately post ECT [19]. This pattern has been described for BIS^™^ values previously [20]. How to explain this finding is unclear at this time but supports the concept that processed EEG data are not useful for consciousness assessment at this stage in the ECT treatment.

Likewise, we discovered a wide range of recovery PSI values between 20 and 88. A significant proportion of patients may have PSI values indicating an anesthetized state when indeed they are clinically awake and able to follow simple commands. A similar observation was reported for BIS^™^ values at this time after ECT [10, 12, 27, 29–31]. These findings suggest a need for further study of these phenomena.

Interestingly, our results showed that right unilateral stimulus delivery resulted in significantly lower mean recovery PSI than bilateral stimulus delivery. However, the sample size for these data is small, and just as aluded to above, an explanation for this finding awaits further study.

Future studies should examine the utility of EEG brain wave monitoring for anesthetic induction agent dosing and possible effects on neuronal depolarization duration and quality. This should include the ability to carry out anesthetic and stimulus dose adjustments during an ongoing ECT treatment cycle and for changes during ECT maintenance therapy.

Our study has multiple limitations that include the retrospective nature of the study, the small sample size, and the participation of a specific patient population, namely men veterans with an advanced average age. We are mindful of a certain degree of missing data. We did not examine stimulus-specific detail or comorbidities when examining the PSI values at various stages during ECT, and we did not account for additional sedation administration after seizure activity and prior to emergence or for the type of neuromuscular blockade used (depolarizing vs non-depolarizing). Due to the nature of the SedLine monitoring system, we were only able to monitor frontal electrical activity. However, our results are intriguing and present an opportunity for future multidisciplinary hypothesis generation, investigation, and possibly improved ECT care for patients in need.

As a practical consideration, during the implementation phase of anesthetic effect monitoring in our ECT suite, two SedLine^®^ machines were damaged most likely as a result of electrical backing into the machine during stimulus delivery. To prevent event, we disconnected the sensor from the monitor during stimulus delivery and immediately reconnected the sensor at the end of the stimulus.

In conclusion, it is clinically feasible to use anesthetic effect monitoring during ECT. Our data provide evidence that the post-stimulus neuronal depolarization time measured by the SedLine^®^ brain function monitoring time is equivalent to the time assessed by the traditional ECT EEG machine. The clinical utility of the SedLine^®^-generated PSI values requires further investigation and is particularly questionable following neurostimulation. Provider training on frontal raw EEG interpretation for conciousness assessment is desirable [32].
